# First Molecular Evidence for the Presence of *Anaplasma phagocytophilum* in Naturally Infected Small Ruminants in Tunisia, and Confirmation of *Anaplasma ovis* Endemicity

**DOI:** 10.3390/pathogens11030315

**Published:** 2022-03-03

**Authors:** Youmna M’ghirbi, Beatriz Oporto, Ana Hurtado, Ali Bouattour

**Affiliations:** 1 Laboratoire Virus, Vecteurs, Hôtes, Service d’Entomologie Médicale, Institut Pasteur de Tunis, Université Tunis El Manar, Tunis 1002, Tunisia; ali.bouattour@pasteur.tn; 2 Department of Animal Health, NEIKER—Instituto Vasco de Investigación y Desarrollo Agrario, Basque Research and Technology Alliance (BRTA), Bizkaia Science and Technology Park 812L, 48160 Derio, Bizkaia, Spain; boporto@neiker.eus (B.O.); ahurtado@neiker.eus (A.H.)

**Keywords:** *Anaplasma phagocytophilum*, *Anaplasma ovis*, small ruminants, duplex PCR assay, Tunisia

## Abstract

*Anaplasma* species are obligate intracellular rickettsial vector-borne pathogens that impose economic constraints on animal breeders and threaten human health. *Anaplasma ovis* and *Anaplasma phagocytophilum* infect sheep and goats worldwide. A duplex PCR targeting the *msp2* and *msp4* genes of *A. phagocytophilum* and *A. ovis*, respectively, was developed to analyze the field blood samples collected from sheep and goats. A total of 263 apparently healthy small ruminants from 16 randomly selected flocks situated in 3 bioclimatic zones in Tunisia were analyzed for *Anaplasma* infections. *Anaplasma* spp. was detected in 78.3% (95% confidence interval (CI): 72.8–83.1) of the analyzed animals. The prevalence of *A. ovis* in sheep (80.4%) and goats (70.3%) was higher than that of *A. phagocytophilum* (7.0% in sheep and 1.6% in goats). Using an inexpensive, specific, and rapid duplex PCR assay, we provide, to the best of our knowledge, the first molecular evidence for the presence of *A. phagocytophilum* in small ruminants in Tunisia. *A. phagocytophilum* generally presented as a co-infection with *A. ovis*. This study provides important data to understand the epidemiology of anaplasmosis in small ruminants, and highlights the risk of contracting the infection upon tick exposure.

## 1. Introduction

The genus *Anaplasma* (order Rickettsiales; family Anaplasmataceae) includes tick-transmitted bacteria with veterinary and human health impacts [1]. *Anaplasma* spp. include ruminant species, such as *Anaplasma ovis*, which is an intraerythrocytic rickettsial pathogen of sheep, goats, and wild ruminants in several zones, including the Mediterranean region of Europe [2,3,4]. In general, the infection of animals with *A. ovis* is asymptomatic [5], however, severe clinical cases caused by this *Anaplasma* species have been reported [6]. Additionally, similar to other *Anaplasma* spp., infection with *A. ovis* may predispose animals to other infections and parasite infestations resulting in clinical disease and eventually death [7]. Recently, *A. ovis* became a potential zoonotic agent since the first documented human case was reported in a young woman with high fever in Cyprus [8].

The *Anaplasma* genus also includes *A. phagocytophilum* (previously known as *Ehrlichia equi*, *Ehrlichia phagocytophila*, and the human granulocytic ehrlichiosis agent), which infects a wide range of hosts, including humans, wild, and domesticated animals, and causes human, canine, and equine granulocytic anaplasmosis as well as tick-borne fever of ruminants [1]. Several mammalian hosts and ticks with persistent infection serve as reservoirs of *A. phagocytophilum* in nature [7].

In Tunisia, several studies detected the presence of various species of *Anaplasma*, such as *A. phagocytophilum* in horses and cattle; *A. marginale*, *A. bovis*, and *A. centrale* in cattle; *A. ovis* and *A. platys*-like in small ruminants; and *A. platys* in camels, dogs, and ticks [9,10,11,12,13,14,15,16]. These studies used several molecular biology tools. Therefore, to improve the diagnosis and the detection of small ruminant’s anaplasmosis, we use in this study a molecular tool based on a single-step duplex PCR for the simultaneous detection and differentiation of *A. ovis* and *A. phagocytophilum* in small ruminants in Tunisia.

## 2. Results

### 2.1. Duplex PCR Assay Performance

From the template plasmids, 420 bp and 334 bp fragments of *Anaplasma ovis* and *A. phagocytophilum*, respectively, were generated. No amplification of the DNA from the uninfected sheep, used as a negative control, was observed. In addition, the DNA of *A. marginale*, *A. platys*, *Ehrlichia* sp., *Ehrlichia canis*, and *Rickettsia conorii* showed no amplification.

When present as single template plasmids, 1 copy of *A. ovis* and 10 copies of *A. phagocytophilum* were detected by the duplex PCR (Table 1). In mixed plasmid combinations, the detection limit was 10 copies for both species, even when the differences in their concentrations were of 2 orders of magnitude. The detection limit was the same when just the plasmid DNA was included and in the presence of uninfected host DNA (spiked controls).

### 2.2. Analysis of the Blood Samples Using the PCR Duplex Assay

Using the duplex PCR, 78.3% (206/263; 95% confidence interval (CI): 72.8–83.1%) of the tested small ruminants was positive to *A. ovis* and/or *A. phagocytophilum* (Table 2). *A. ovis* was detected in 77.9% (205/263; 95% CI: 72.4–82.8%) of analyzed animals with 80.4% (160/199; 95% CI: 74.2–85.7%) infected sheep and 70.3% (45/64; 95% CI: 57.6–81.1%) infected goats (Table 2). The difference of *A. ovis* infection between sheep and goats was not significant (*p* = 0.54). Similarly, no significant difference of *A. ovis* small ruminant infection rates (*p* = 0.08) was observed within the 3 bioclimatic zones: humid (72.7%; 95% CI: 64.6–79.8%), sub-humid (89.8%; 95% CI: 80.2–95.8%), and semi-arid (76.5%; 95% CI: 62.5–87.2%). The prevalence of *A. ovis* in sheep flocks ranged from 53.8% (95% CI: 25.1–80.8%) to 100% (95% CI: 82.3–100%), with no significant difference (*p* = 0.09) among the 3 bioclimatic zones. Similarly, the goat’s prevalence in the different bioclimatic zones ranged from 54.3% (95% CI: 36.6–71.2%) to 100% (95% CI: 69.1–100%), with no significant difference (*p* = 0.58) in the 3 bioclimatic zones. In addition, no significant difference (*p* = 1.3) was recorded between *A. ovis*-infected adults (82.8%; 183/221; 95% CI: 77.2–85.5%), and infected lambs and kids (22/42; 52.4%; 95% CI: 36.4–68.0%).

Single infection by *A. phagocytophilum* was detected in 1 adult sheep (0.4%; 95% CI: 0.01–2.1%). Otherwise, *A. phagocytophilum* was detected in combination with *A. ovis* in 5.3% (14/263; 95% CI: 2.9–8.7%) of animals (Table 2), i.e., 13 adult sheep (7%; 95% CI: 4–11.5%) from the 3 investigated bioclimatic zones and 1 adult goat from the semi-arid zone (1.6%; 95% CI: 0.04–8.4%).

## 3. Discussion

Several molecular biology techniques were proposed for the identification of Anaplasmataceae species. Most of them target the heat shock gene groEL [17], the 23S rRNA [18], the 16S rRNA gene [19], and the major surface proteins (MSPs) [3]. In this study, using the *msp*4 and *msp*2 gene sequences, we successfully developed a specific and rapid duplex assay to differentiate *A. phagocytophilum* and *A. ovis* that does not require sophisticated laboratory equipment. Indeed, this optimized duplex PCR has the capability to specifically detect *A. phagocytophilum* and *A. ovis* from both single and mixed infections. The detection limit of the duplex PCR was the same for both species when present as a mixture (10 copies), but the sensitivity of the assay was better for *A. ovis* (1 copy) than for *A. phagocytophilum* (10 copies) for single infections. Moreover, our results show that the sensitivity of the PCR was the same in a single and a duplex format. This new assay adds to a similar one developed for the identification of the main *Anaplasma* species that infect cattle; by using the reverse primer designed here for *A. ovis* instead of the one for *A. marginale* (M4-Mar-R), the assay can be transformed to be used for small ruminants [20].

This study is the first report of the presence of *A. phagocytophilum* in small ruminants in Tunisia. However, previous studies, using molecular tools, detected *A. phagocytophilum* in cattle [20], dogs, horses, and ticks [14,15,16]. Our investigation shows that small ruminants in Tunisia were infected by *A. ovis* and *A. phagocytophilum*. The overall prevalence of *Anaplasma* spp. in small ruminants (78.3%) was higher than that reported in Italy (42%) [21], China (42%) [22], Iran (34%) [23], and Turkey (46.6%) [24]. In fact, anaplasmosis is widely distributed throughout the world and the prevalence rates vary according to the region and the analysis technique used [25].

The average prevalence rate of *A. ovis* in sheep (78%) was almost similar to that reported in other Tunisian regions (70–93.8%) [13] and also in other countries, such as Portugal (82.5%) [26]. In contrast, this rate was higher than those reported in other sites from northern Tunisia (35.6%) [11], China (40.5%) [22], Sudan (41.7%) [27], Turkey (31.4%) [26], and Iran (44%) [23]. The study herein did not find significant differences in the prevalence of *A. ovis* in sheep (80.4%) and goats (70.3%). In contrast, using PCR targeting 16S rRNA gene, Belkahia et al. [11] reported lower prevalence in other regions in Tunisia (35.6%). In addition, lower prevalences were reported in China (15.3–25.6%) [28,29], and recently in Turkey (45.7% in sheep and 50% in goats) [24]. In fact, this high *A. ovis* prevalence rate fits well with our previously data, which reported that 95.5% of ticks collected from ruminants in the studied regions were *Rhipicephalus turanicus* [30], the tick species considered the vector of *A. ovis*. Indeed, DNA of *A. ovis* was detected in most of this and other *Rhipicephalus* species in Tunisia [31], Italy [32], Algeria [33], and France [34]. In addition, several other tick species, belonging to *Rhipicephalus*, *Hyalomma*, and *Dermacentor* genera, were previously reported as probable vectors of *A. ovis* worldwide [35,36]. Likewise, other arthropod species can be involved in the transmission of *A. ovis* [37]. In this context, the role of the biting hematophagous insects, such as Hippoboscidae flies [38,39], infesting sheep and occasionally goats, and some species of fleas [40] should not be underestimated with regard to the transmission of anaplasmosis.

The differences in the prevalence rates of *A. ovis* in small ruminants recorded in the different regions may be due to several factors, such as the presence and the abundance of ticks, methods used for sample analysis, wildlife reservoir presence [32], the management of farms and husbandry practices, bioclimatic and ecological parameters, and susceptibility of host species and breeds. In addition, goats are known to spend less time grazing and graze just within the home boundaries compared to sheep that graze far into the bush, hence coming into contact with vegetation and subsequently more ectoparasites.

*Anaplasma phagocytophilum* was detected in small ruminants in Tunisia for the first time. However, it was less frequently detected in sheep in this study compared to studies carried out in Northern Slovakia [41], China [42], and Turkey [43]. This low prevalence could be explained by (i) the low number of intragranulocytic *A. phagocytophilum* circulating in carriers animals [44]; (ii) the low tick infection rate by *A. phagocytophilum*; and (iii) the short duration of *A. phagocytophilum* bacteremia during the acute phase of infection [45,46,47]. In Tunisia, *A. phagocytophilum* was reported in the humid and sub-humid investigated zones in which *Ixodes ricinus*, the main vector of *A. phagocytophilum* in Europe [48,49], occurred [50].

Interestingly, *A. phagocytophilum* was detected in three small ruminants from the semi-arid zone in which *Ixodes icinus* is absent, suggesting that this bacterium is probably maintained in foci by other Ixodidae. Indeed, Estrada-Peña [51] reported that other Ixodidae species of the genera *Hyalomma*, *Rhipicephalus*, and *Haemaphysalis* are potential vectors of *A. phagocytophilum*. In the investigated semi-arid regions, these tick genera heavily infested ruminants [50,52]. These results support the idea that several tick species may maintain or be involved in the transmission of *A. phagocytophilum*.

In this study, we observed mixed infections with *A. phagocytophilum* and *A. ovis* in sheep and goats, which suggests that Tunisia is a country with endemic occurrence of pasture fever, gradually spreading from humid to semi-arid sites. This agreed with the results reported in southeastern and northern Slovakia, but contradicted the study carried out in northwestern China [53]. In Italy, [21] a high occurrence of mixed infection with *A. phagocytophilum* and *A. ovis* was detected in a sheep flock with health issues. Similar results were recorded in Slovakia [54], Turkey [24], Germany [55], and China [56]. A description of the coinfection of small ruminants in Tunisia with more than one pathogen has already been reported [30]. Coinfection favors health problems of the animals and can consequently increase the loss of productivity [57]. Authors speculated that immunosuppressed animals with poor health conditions are more vulnerable to the multiple *Anaplasma* infections. Finally, *A. phagocytophilum* and *A. ovis* circulate within small ruminant populations across Mediterranean countries, but the extent of infection seems to vary among countries. These variations may be related to climate conditions, different husbandry systems, and the occurrence of these species in the tick population.

## 4. Materials and Methods

### 4.1. Design of Primers

To design the primers, Vector NTI 8.0 software (Informax Inc., North Bethesda, MD, USA) was used to align *A. ovis* *msp*4 and *A. phagocytophilum* *msp*2 gene sequences with those of other *Anaplasma* and *Ehrlichia* species. Then, a new reverse primer (M4-Ov-R: 5′-ATGTCCTTGTAAGACTCGTCAAAGAGT-3′) was designed to be used with the forward primer M4-OvMar-F described elsewhere [20], in order to specifically amplify a 420 bp fragment of the *msp*4 gene of *A. ovis.* These primers were used in combination with the previously designed primers that amplify a 334 bp fragment of the *msp*2 gene of *A. phagocytophilum* [58].

### 4.2. Cloning and Sequencing the msp4 A. ovis Gene and the msp2 A. phagocytophilum Gene

DNA extracted from two naturally infected sheep with *A. ovis* and *A. phagocytophilum* using QIAamp DNA Mini Kit (QIAGEN, Hilden, Germany) as per the manufacturer’s recommendations was used as a template to amplify a 420 bp and 334 bp of *msp*4 and *msp*2 genes, respectively. A pCR4-TOPO vector was used to clone the amplified products according to the manufacturer’s instructions (TOPO TA cloning kit for sequencing; Invitrogen, Carlsbad, CA, USA). Recombinant plasmid DNA was purified using a FlexiPrep kit (Amersham Biosciences, Freiburg, Germany), subjected to automatic dye terminator cycle sequencing, and the nucleotide sequences of the plasmid inserts were confirmed as *A. phagocytophilum* and *A. ovis* using Blast (https://blast.ncbi.nlm.nih.gov/Blast; accessed on 30 June 2019).

The plasmid concentrations were controlled using a NanoDrop ND-1000 spectrophotometer (Thermo Scientific, Germany) and the plasmids were 10-fold serially diluted from 10^8^ to 10 copies/µL in a Tris-EDTA buffer. The sensitivity of the assay was tested using serial dilutions of individual plasmids as well as different combinations of both plasmids.

### 4.3. Duplex PCR Amplification

A commercially available Multiplex-PCR assay kit (QIAGEN, Hilden, Germany) was used to perform PCR reactions in 25 μL volume reactions, including 1× QIAGEN Multiplex PCR Master Mix (QIAGEN, CA), 0.5 μM of Msp2-3F/Msp2-3R primers, 0.2 µM M4-OvMar-F/M4-Ov-R primers, and 10 to 50 ng/μL of extracted DNA. The qPCR program was the following: 15 min at 95 °C, followed by 40 cycles at 94 °C for 30 s, 63 °C for 90 s, 72 °C for 90 s, and a final step at 72 °C for 10 min. To avoid false-positive reactions and cross-contaminations, PCR reactions were set up in a separate room, plugged tips were used, and a negative (water) control was included in each run.

### 4.4. Analysis of the Sensitivity and Specificity of Single and Duplex PCR Assay

Ten-fold serial dilutions of individual plasmids with the two inserts of *A. phagocytophilum* and *A. ovis* as well as different combinations were amplified, as described above, to determine the detection limit of the single and the duplex PCR assays. In addition, sensitivity was tested using an extracted DNA from blood of a non-infected sheep spiked with the same plasmid combinations. DNA from other Rickettsiale species (*Anaplasma marginale*, *Anaplasma platys*, *Ehrlichia* sp., *Ehrlichia canis*, and *Rickettsia conorii*) were used to test the specificity of the PCR assays.

### 4.5. Study Sites, Blood Sampling, and DNA Extraction

This cross-sectional study was conducted in 2015 in 9 localities (Table 2) situated in 3 different bioclimatic zones (humid, sub-humid, and semi-arid) in northern Tunisia (Table 2). All sites have a Mediterranean climate: cool, moist winters and dry, hot summers. Sheep and goat flocks were randomly chosen following the recommendations of the State Veterinary Office as representative of the local traditional management system, with small flocks grazing on permanent pastures or bush. Animals (*n* = 263) were selected from 16 flocks, including 199 Barbarine sheep (193 females and 6 males) and 64 goat females of local breed (Table 2). Among these animals, 42 were younger than 1 year (26 lambs, 16 kids) and 221 were adults (173 sheep, 48 goats). These small ruminants were bled once between April and June 2015 during the season of tick activity.

DNA was extracted from the blood using the PureLink Genomic DNA Kit for DNA purification (Invitrogen, Carlsbad, CA, USA) and yields were determined using a NanoDrop ND-1000 Spectrophotometer (Thermo Scientific, Dreieich, Germany). Extracted DNA was tested for the presence of *A. ovis* and *A. phagocytophilum* using the duplex PCR assay as described above. Amplified fragments were subjected to electrophoresis on a 1.5% agarose gel stained with GelRed Nucleic Acid Gel Stain (Biotium, Koln, France), and then visualized by UV transillumination.

### 4.6. Statistical Analysis

The chi-squared or Fisher’s exact tests were used to compare the proportions of positivity by the host (sheep and goats) in the three bioclimatic zones and age groups (adult vs. lamb/kid). The *p*-values of 0.05 or less were considered statistically significant.

## 5. Conclusions

We presented here the first molecular evidence for the presence of *A. phagocytophilum* in naturally infected small ruminants in Tunisia, and confirmed that *A. ovis* is endemic in the different bioclimatic zones of Tunisia. The occurrence of *A. phagocytophilum* in small ruminants was rare and it generally presented as a mixed infection with *A. ovis*. These results deserve more attention and highlight the need to investigate other farming regions to understand the epidemiology of anaplasmosis, since *A. phagocytophilum* prevalence is certainly underestimated in Tunisia. In addition to the threats that granulocytic anaplasmosis might pose to livestock in Tunisia, *A. phagocytophilum* and *A. ovis* can have adverse effects on human health. Therefore, the zoonotic potential of these species should no longer be neglected, especially as the epidemiology of human granulocytic anaplasmosis is still unknown in North Africa. Furthermore, the investigation of their clinical impact, particularly in case of comorbidities and of potential tick vectors is useful to understand the life cycle and promote a One Health approach to prevent and control the infection.

## Figures and Tables

**Table 1:** Duplex PCR assay sensitivity test: results of the amplification of different plasmid combinations and the DNA extracted from the blood of a non-infected sheep spiked with the same plasmid combinations.

Plasmid Copies ^a^	DNA Uninfected Sheep ^b^	*Anaplasma ovis*	*Anaplasma phagocytophilum*
10^3^ Ap + 10 Ao	P	Positive	Positive
10^3^ Ap + 10 Ao	A	Positive	Positive
10 Ap + Ao 10^3^	P	Positive	Positive
10 Ap + Ao 10^3^	A	Positive	Positive
10 Ap	P	Negative	Positive
10 Ap	A	Negative	Positive
1 Ap	P	Negative	Negative
1 Ap	A	Negative	Negative
10 Ao	P	Positive	Negative
10 Ao	A	Positive	Negative
1 Ao	P	Positive	Negative
1 Ao	A	Positive	Negative

^a^: Ao, plasmid with an insert of the *msp4* gene fragment of *Anaplasma ovis*; Ap, plasmid with an insert of the *msp2* gene fragment of *Anaplasma phagocytophilum*. ^b^: P, presence in the PCR reaction of DNA extracted from the blood from a non-infected sheep spiked with the indicated plasmid or plasmid combinations; A, absence of host DNA, water was used instead.

**Table 2:** Duplex PCR detection and identification of *Anaplasma ovis* and *Anaplasma pahgocytophilum* in small ruminants in Northern Tunisia.

Bioclimatic Zone	Localities (*n* Animals: Sheep: S, Goat: G)	Latitude; Longitude	Sheep (*n* = 199)		Goats (*n* = 64)	
			*A. ov* (%)	*A. pha* (%)	*A. ov* (%)	*A. pha* (%)
Humid	Tabarka (17S, 4G)	36.93557; 8.76174	12 (70.6)	1 (5.8)	3 (75.0)	0
	Amdoun (24S)	36.76783; 9.07399	18 ^a^ (75.0)	1 ^a^ (4.2)	ns	ns
	Sejnene (12S, 35G)	37.13415; 9.26011	10 (83.3)	0	19 (54.3)	0
	Maden (28S, 10G)	36.96668; 9.08898	25 (89.3)	0	10 (100.0)	0
	Nefza (13S)	36.98083; 9.08416	7 ^b^ (53.8)	2 ^b^ (15.4)	ns	ns
Total Humid (94S, 49G)			72 (76.6)	4 (8.2)	32 (65.3)	0
Sub-Humid	Oued El Abid (19S, 1G)	36.86625; 10.74154	19 ^c^ (100.0)	3 ^c^ (15.8)	1 (100.0)	0
	Mellegue (11S)	36.25407; 8.57642	10 ^d^ (90.9)	5 ^d^ (45.5)	ns	ns
	Touiref (38S)	36.34741; 8.59298	32 (84.2)	0	ns	ns
Total Sub-Humid (68S, 1G)			61 (89.7)	8 (11.8)	1 (100.0)	0
Semi-Arid	El Jouf (37S, 14G)	36.31305; 10.10583	27 ^b^ (73.0)	2 ^b^ (5.4)	12 ^e^ (85.7)	1 ^e^ (7.1)
Total Semi-Arid (37S, 14G)			27 (73.0)	2 (5.4)	12 (85.7)	1 (7.1)
Total			160 (80.4)	14 (7.0)	45 (70.3)	1 (1.6)

Superscripts denote the mixed infections with *A. ovis* and *A. phagocytophilum*: ^a^ in 1 sheep; ^b^ in 2 sheep; ^c^ in 3 sheep; ^d^ in 5 sheep; ^e^ in 1 goat; ns; not sampled.

## Data Availability

Not applicable.
